# P-1716. Assessing Appropriateness of Cephalosporins Prescription in a Tertiary Care Hospital in South India

**DOI:** 10.1093/ofid/ofae631.1881

**Published:** 2025-01-29

**Authors:** Rithik Dharan, Suresh Kumar Dorairajan, Suhail Hassan, M Roshni

**Affiliations:** The Tamil Nadu Dr. MGR Medical University, Chennai, Tamil Nadu, India; Apollo hospital, Chennai, Tamil Nadu, India; Tamilnadu Dr. MGR University, Chennai, Tamil Nadu, India; Dr.MGR medical University, Chennai, Tamil Nadu, India

## Abstract

**Background:**

Cephalosporins plays a vital role in empirical therapy. Unfortunately the misuse & underuse of 3rd Generation Cephalosporins (3GCs) has triggered a concerning rise in ESBL-producing Enterobacterales, with resistance rates as high as 80-85% in India. If this ongoing misuse continues, it poses a serious threat to the efficacy of these vital antibiotics. This cross-sectional study aims to evaluate the appropriateness and utilization of Cephalosporins in a tertiary care hospital in South India.

**Table 1:**
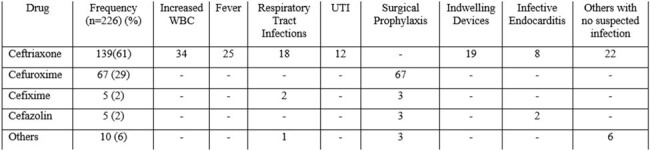
Factors for the initiation of Cephalosporins

**Methods:**

In April 2024, a cross-sectional study was conducted by Pharm D interns assisted with Infectious Disease Specialists. Patients admitted as IP who received Cephalosporins until discharge are included. Patients receiving other than Cephalosporins are excluded from the study. Therapy appropriateness was determined using the Sanford Antimicrobial Guide 2022. Simple statistics are done in Excel for analysis.

**Table 2:**
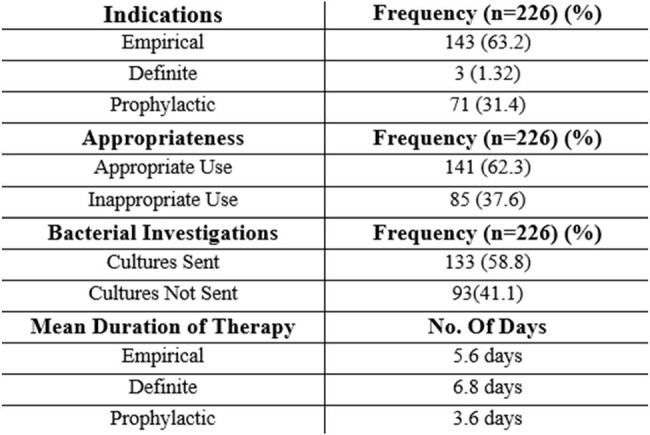
Assessment of the initiated Cephalosporins

**Results:**

Of 1376 patients, 428 (31.1%) patients received antimicrobial therapy of that Cephalosporins were prescribed to 226 patients (52.8%) for various indications. The most commonly prescribed was Ceftriaxone 139 (61.5%) prescriptions, followed by Cefuroxime 67 (29.6%), Cefixime 5 (2.2%), Cefazolin 5 (2.2%) & others 10 (4.4%). Reasons for initiating cephalosporins and analysis are given in Table 1 & Table 2. 3GCs are the most frequently used empirical antibiotic. A significant proportion (37.6%) of cephalosporin prescriptions lacked clinical infections. Empirical therapy continued beyond the 5th day 5.8 ± days following negative cultures, Duration of surgical prophylaxis exceeded than the recommended guidelines.

**Conclusion:**

From the findings, Ceftriaxone dominated 61% of prescriptions for various indications, with one-third administered inappropriately via parenteral till discharge, mean duration 5.8 ± days. 37.6 % of the 3GC administered have been inappropriate also which has added additional costs to the patient. A knowledge gap among clinicians in discontinuation should be addressed by educational interventions with clinical pharmacists actively engaging to de-escalate cephalosporins. This single-centric study suggests the need for RCTs providing alternatives to cephalosporins for infections to prevent the risk of increasing ESBL resistance in India.

**Disclosures:**

**All Authors**: No reported disclosures

